# Pectoralis Minor Tenotomy with Occasional Secondary Neurolysis Significantly Reduces Self-Reported Pain and Headaches Across Heterogenous Chronic Pain Disorders of the Upper Limb

**DOI:** 10.3390/medicina62061071

**Published:** 2026-06-01

**Authors:** Ketan Sharma, James M. Friedman

**Affiliations:** 1 St. Luke’s Plastic and Reconstructive Surgery, Boise, ID 83712, USA; 2 Sutter Alpine Care Clinic, Stockton, CA 95204, USA; james.friedman46@gmail.com

**Keywords:** chronic pain, upper limb, scapula, pectoralis minor

## Abstract

*Background and Objectives*: Many patients suffer from chronic pain of the shoulder, neck, upper back, and/or arm. They may be diagnosed with fibromyalgia, complex regional pain syndrome, myofascial pain, thoracic outlet, subacromial pain, cervical radiculopathy, cervicogenic headaches, post-mastectomy pain, and/or occupational shoulder disorder. The pectoralis minor (PM) is the only muscle of the scapula controlled by the lower trunk of the brachial plexus. In the Human Disharmony Loop (HDL), this neurologic asymmetry produces persistent protraction of the scapula. Protraction deforms the scapula’s connections, generating headaches and neck stiffness, upper back tightness, shoulder weakness, and hand numbness. We hypothesize patients with the above who meet HDL diagnostic criteria will benefit from PM tenotomy with brachial plexus neurolysis (PM+ICN). *Materials and Methods*: Patients diagnosed with the above disorders who also met HDL criteria of medial coracoid tenderness and scapula protraction on exam underwent PM+ICN, with secondary neurolysis after 3 months if needed. Clinical neuropathy was diagnosed via the scratch-collapse test. Outcomes included self-reported Visual Analogue Score pain scores, active shoulder abduction range of motion (ROM), prevalence of occipital headaches. *Results*: *N* = 318 patients were included. Average age was 51; 68.0% were female. Following treatment, average pain decreased from 7.3/10 to 2.1/10 (*p* < 0.001), average shoulder ROM increased from 96 to 170 degrees (*p* < 0.001), and occipital headaches decreased from 76.7% to 1.6% (*p* < 0.001). Scapular protraction normalized from 98.8% static to 92.5% none (*p* < 0.001). Overall, 17% required subsequent neurolysis, chiefly of the axillary, radial, and ulnar nerves. The pain reductions were statistically indistinguishable across all diagnoses (*p* = 0.709, I^2^ = 0.02%). Average follow-up was 22 months. *Conclusions*: PM+ICN significantly reduced self-reported pain and headaches in select intractable patients. The PM pathologizing the scapula may constitute a shared anatomic mechanism that contributes to chronic pain across heterogenous disorders of the upper limb.

## 1. Introduction

Humans are plagued by chronic pain. As the most common disability worldwide, it consumes over $650 billion in annual costs [1]. One-fifth of Americans suffer from chronic pain at any given time, of which two-thirds persist longer than a year [2]. Upper back and neck pain alone constitutes the fourth leading cause of disability [3]. Despite its ubiquity, chronic pain afflicting the upper limb girdle—neck, upper back, shoulder, and arm—remains mysterious and challenging. This includes syndromes such as thoracic outlet (TOS), scapular dyskinesis (SD), myofascial pain (MPS), fibromyalgia, complex regional pain syndrome (CRPS), work-related musculoskeletal disorder (WRMD), post-mastectomy pain syndrome (PMPS), cervical radiculopathy, subacromial pain (SAPS), and cervicogenic headaches. These feature some manifestation of pain—headaches, stiffness, tightness, weakness, numbness, and/or coolness—from the neck to the fingertips. They also share contentious diagnostic criteria, convoluted pathophysiology, and sometimes ineffectual treatments [4,5,6,7,8,9,10,11]. Chronic pain regrettably remains a disease with end goals of treatment and not cure [12].

The key to the upper limb is the scapula [13]. The scapula dynamically connects the body (thorax) to the arm (humerus), coordinating mobility and stability. To enable the full arc of overhead reach, the scapula glides along the thorax to perform two critical functions: optimizing excursion of the deltoid and rotator cuff and maintaining articular congruity to prevent impingement. The peri-scapular chain, comprising the dorsal trapezius, levator scapulae, rhomboids, lateral serratus anterior, and ventral pectoralis minor (PM), controls this motion. Given its central role, abnormal scapulothoracic motion or SD can generate a wide array of dysfunction [14], but has numerous proposed causes which are more likely associations rather than etiologies [15].

**Figure 1:**
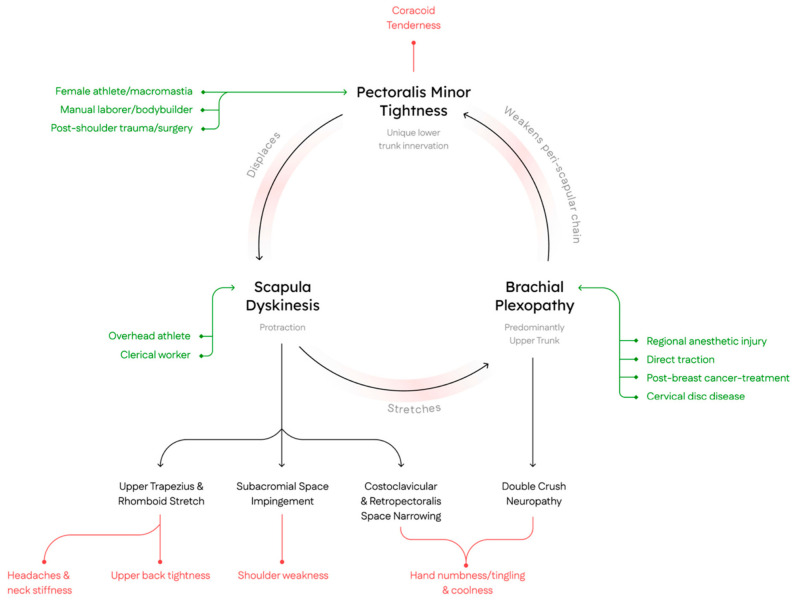
The human disharmony loop. The central loop has three elements. Diverse patients can enter via each element (green). The pathoanatomic sequelae produce four groups of symptoms (bottom row, red). The positive feedback nature may contribute to intractable pain of the human upper limb.

**Figure 2:**
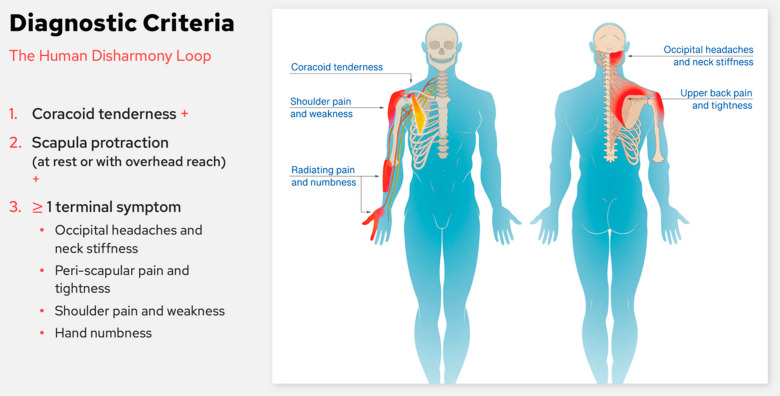
Human disharmony loop clinical presentation diagnoses derived from two physical exams and one history criteria. (+ indicates that all three criteria are required).

**Figure 3:**
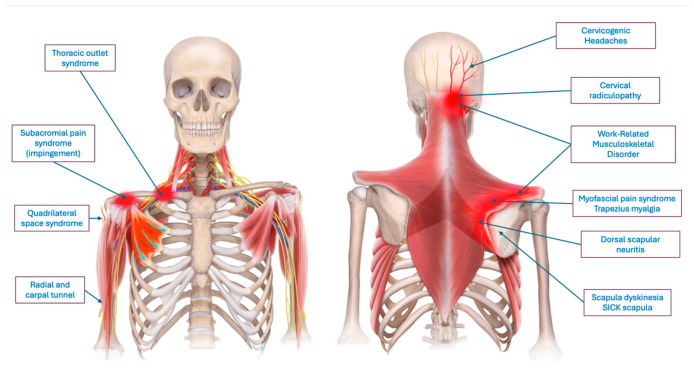
Human disharmony loop pathoanatomy linking to chronic pain syndromes. Theoretically, the PM (orange, left) protracting the scapula and deforming its ventral and dorsal connections anatomically connects to various upper limb chronic pain syndromes.

Previously, we described the Human Disharmony Loop (HDL), a clinical model of upper limb dysfunction [15,16,17] (Figure 1). A neurologic asymmetry surrounds the scapula that may be a sequelae of our evolution from quadrupeds to bipeds: the upper C4-6 roots of the brachial plexus control the dorsal peri-scapular chain, while the lower C8-T1 trunk controls the ventral PM [15]. This asymmetry can lead to persistent protraction of the scapula, the direction of pull by the PM. Protraction deforms the scapula’s numerous connections, producing occipital headaches and neck stiffness, upper back tightness, shoulder weakness, and hand numbness/tingling [16] (Figure 2). Treatment consists of PM tenotomy with infraclavicular plexus neurolysis (PM+ICN), with secondary neurolysis if needed, to normalize scapular mechanics and reharmonize the limb girdle. Theoretically, the HDL anatomically connects to the above chronic pain disorders (Figure 3). Hence, we hypothesize the PM pathologizing scapular mechanics may contribute to refractory symptoms in these conditions. In this paper, we report the outcomes of PM+ICN to treat chronic upper limb pain in patients who meet HDL criteria.

## 2. Materials and Methods

This is a retrospective, single-center case series of consecutive patients presenting with chronic pain treated by a single fellowship-trained, board-certified hand surgeon, between January 2023 and October 2025. Inclusion criteria consisted of (1) age > 13 years; (2) a diagnosis of any of the following made by the referring provider: TOS, SICK scapula/SD, MPS, fibromyalgia, CRPS, WRMD, SAPS, cervical radiculopathy, cervicogenic headaches, PMPS, and Burner/Stinger; (3) symptoms >6 months duration refractory to all other treatments; and (4) diagnosis of HDL. This was determined via coracoid tenderness to palpation with visible scapular protraction on exam and at least one terminal symptom (Figure 2). Exclusion criteria included: follow-up <12 weeks. Clinical neuropathy was identified on exam via the scratch-collapse test (SCT) at the thoracic outlet, PM, suprascapular notch, quadrilateral space, and radial, cubital, and carpal tunnels [18]. All patients trialed at least 6 weeks of therapy before surgery and received an independent evaluation by a certified hand therapist (CHT) confirming all exam findings including coracoid tenderness, scapular protraction, and SCT+ neuropathic lesions. Patients were evaluated pre-operatively and at 2, 6, 12, 24, and 52 weeks post-operatively. At each visit, patients prospectively completed a self-reported Visual Analogue Scale (VAS) pain questionnaire, and active shoulder abduction range of motion (ROM) values were measured. Scapula dyskinesis on exam was classified as none (no visible protraction), dynamic (visible protraction with overhead reach only), or static (visible protraction at rest) [17].

Each patient underwent PM+ICN via an open deltopectoral approach as previously described [15]. This was followed by a standardized PT protocol consisting of brachial plexus and axillary nerve glides at week 2 and medial rhomboid and upper trapezius strengthening with scapula retraction postural taping at week 6 [15,17]. At 12 weeks post-operatively, patients were offered secondary neurolysis for residual pain or weakness if they exhibited a positive SCT at a compressive lesion. Outcomes included self-reported VAS pain scores out of 10, presence of occipital headaches, clinical neuropathy, active shoulder ROM, need for secondary neurolysis, and surgical complications. Outcomes from the most recent visit were used for all analysis. Institutional Review Board (IRB) approval was obtained by the St. Luke’s Health System (14 October 2024, IRB protocol # 2024-0107), and need for consent was waived as the data was anonymous and posed minimal risk to patients.

Continuous variables were reported as mean (95% confidence intervals), while categorical were reported as counts (percentages). Paired Student’s *t*-test and paired McNemar’s test compared continuous and binary categorical variables of interest, respectively, while the marginal homogeneity test of Stuart–Maxwell compared the three-level scapular dyskinesis classification. To assess the cross-group consistency of pain reduction between the diagnoses, Cochran’s Q and I^2^ statistics were applied to the inverse-variance-weighted within-group mean. Two-tailed α = 0.05 was used for all tests, with the Holm-Bonferroni step-down procedure applied for multiple paired comparisons. As this study is retrospective, no a priori sample-size calculation was performed. Post hoc, with 318 paired observations, the study had >99% power to detect a paired effect of Cohen’s d_m_ = 0.20. This study conforms to the STROBE guidelines.

## 3. Results

*N* = 318 patients were included. Mean age was 51.0 years (49.2, 52.9). Sex was 217 (68.0%) female. Prevalence of chronic pain diagnoses were: 53 (16.7%) TOS, 73 (23.0%) fibromyalgia, 45 (14.2%) CRPS, 60 (18.9%) WRMD, 106 (33.3%) MPS, 104 (32.7%) cervical radiculopathy, 266 (83.6%) SAPS, 13 (4.1%) SD, 11 (3.5%) PMPS, and 244 (76.7%) cervicogenic headaches (Table 1). Patients presented with a mean of 3.10 (2.96, 3.23) diagnoses. Three months following PM+ICN, prevalence of clinical neuropathy decreased as follows: thoracic outlet 54.7% to 2.2% (*p* < 0.001), suprascapular notch 57.5% to 1.6% (*p* < 0.001), quadrilateral space 81.4% to 16.0% (*p* < 0.001), radial tunnel 70.1% to 20.4% (*p* < 0.001), and carpal tunnel 41.8% to 16.0% (*p* < 0.001); but it was unchanged at the cubital tunnel, 21.4% to 20.1% (*p* = 0.650). Overall, 17.3% of patients required secondary neurolysis, most commonly of the radial (9.7%), ulnar (10.4%), and axillary (9.4%) nerves.

**Figure 4:**
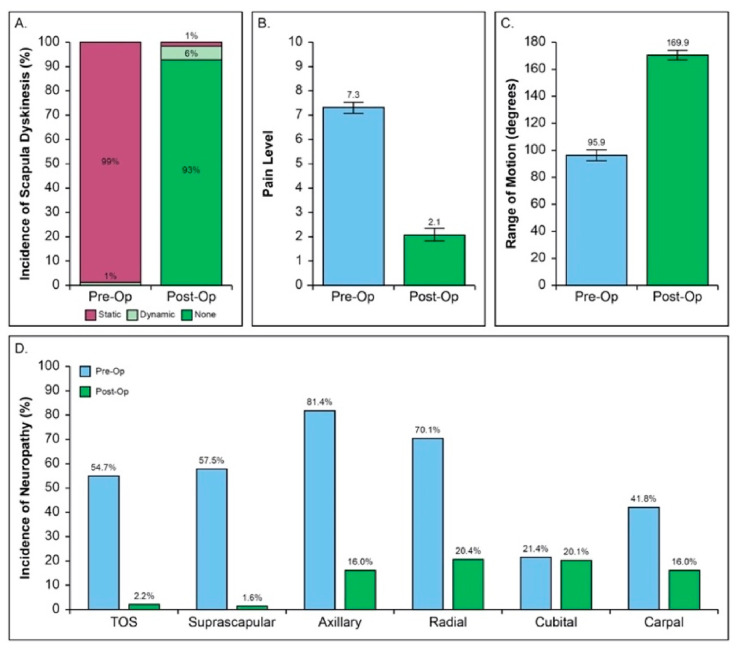
Clinical outcomes. (**A**) PM+ICN normalized scapular mechanics. (**B**) This significantly reduced self-reported pain. (**C**) And increased average shoulder abduction. (**D**) And decreased incidence of concomitant neuropathy at all locations except for the cubital tunnel.

**Figure 5:**
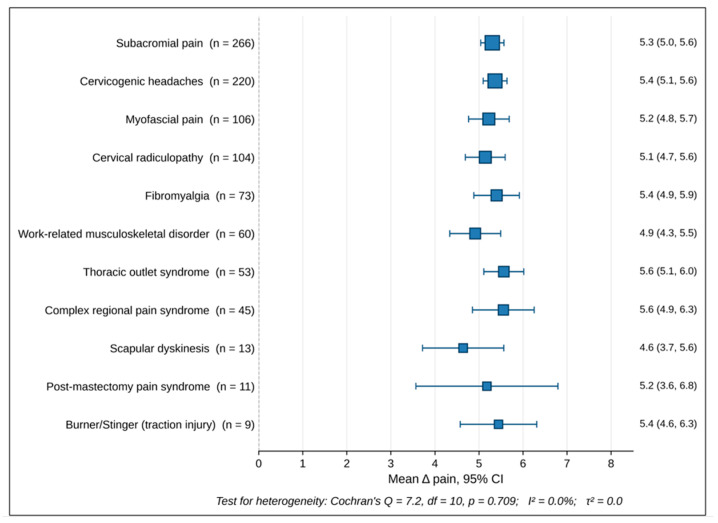
Pain reductions by chronic pain diagnosis. The absolute pain reduction among the heterogenous chronic pain diagnoses was statistically similar, consistent with a shared underlying mechanism.

At most recent follow-up, VAS pain decreased from 7.3/10 to 2.1/10 (*p* < 0.001), headache incidence decreased from 76.7% to 1.6% (*p* < 0.001), and average shoulder abduction increased from 96 to 170 degrees (*p* < 0.001). Scapular dyskinesis normalized from 98.8% static and 1.3% dynamic pre-operatively to 92.5% none, 5.7% dynamic and 1.3% static post-operatively (*p* < 0.001) (Figure 4). Mean follow-up was 22 months. There were 11 (3.5%) complications, most commonly seroma 4 (1.3%) or wound dehiscence 2 (0.6%) (Table 2). The reductions in pain were statistically indistinguishable across all diagnoses (Cochran’s Q = 7.2, df = 10, *p* = 0.709, I^2^ = 0.02%) (Figure 5).

## 4. Discussion

**Figure 6:**
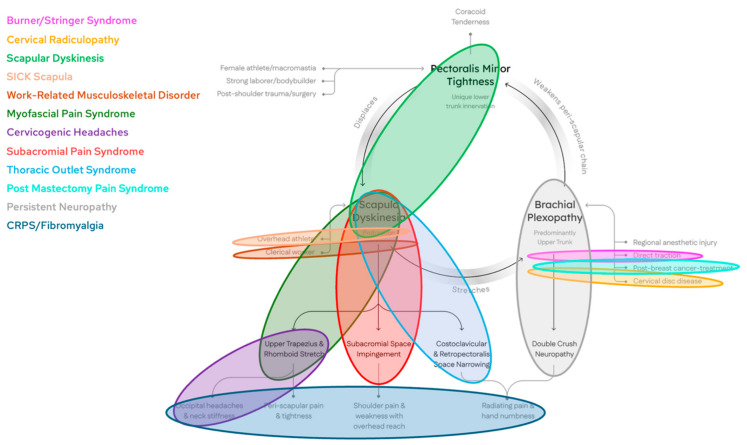
Theoretical human disharmony loop shared anatomic mechanism. Theoretically, the HDL may constitute a shared anatomic mechanism that connects the heterogenous chronic pain disorders of the upper limb.

In this study, refractory patients suffering from chronic pain of the upper limb who met HDL criteria underwent PM+ICN. Diagnoses included TOS, SD, MPS, fibromyalgia, CRPS, WRMD, SAPS, PMPS, cervical radiculopathy, and cervicogenic headaches. Despite best efforts, these remain challenging, with contentious diagnosis, convoluted pathophysiology, and occasionally ineffective treatments [4,5,6,7,8,9,10,11]. However, normalizing scapular mechanics produced substantial clinical improvement, generating a mean absolute pain reduction of >5 points, more than double the minimum clinically important difference (MCID) of 2 [19]. Furthermore, we observed a uniform response to PM+ICN across a wide range of heterogenous disorders—reducing pain in fibromyalgia, eliminating headaches in cervical radiculopathy, and restoring motion in SAPS, etc. This suggests the PM pathologizing the scapula’s connections may constitute a shared anatomic mechanism that contributes to some of the intractable symptoms seen in these disorders (Figure 6).

The PM pathologizes the full upper limb girdle in several ways. First, PM tightness produces medial coracoid tenderness. Second, brachial plexus traction generates secondary neuropathy via double crush [20]. Third, scapula displacement stretches the upper trapezius and rhomboids [21]. Fourth, upper trapezius stretch irritates the occipital nerves to the scalp [22,23,24]. Fifth, narrowing of the subacromial and costoclavicular spaces compresses the neurovascular bundle and impinges the rotator cuff [25]. (Visually, the shoulder assumes the ubiquitous hunched posture.) Thus, a single anatomic source produces a reproducible regional distribution of pain: occipital headaches and neck stiffness, upper back tightness, shoulder weakness, and hand numbness/tingling (Figure 2).

To date, a variety of specialists have treated these symptoms separately within the context of heterogenous diagnoses [4,5,6,7,8,9,10,11]. However, several lines of evidence suggest these disorders are anatomically linked. First, they occur concomitantly [26], which is corroborated in this study, where patients presented with ~3 separate diagnoses on average. Second, each disorder may feature symptoms outside of their described pathophysiology but within another’s, such as occipital headaches with TOS [4] or upper back tightness with PMPS [27]. Third, in this study, the reductions in pain were remarkably consistent across diagnoses (Figure 5), which provides strong statistical evidence for some degree of shared mechanism. The HDL may constitute this common anatomic link (Figure 3). Theoretically, accounting for the mechanics of the scapula with the protracting pull from the PM elucidates many well-known but unexplained symptoms and observations (Table 3).

For instance, in MPS, the distribution of trigger points is reproducibly localized to the upper trapezius and rhomboid [28]. These two muscles are the principal antagonists to the PM in the peri-scapular chain and therefore undergo pathologic stretch with overpowering PM pull. Similarly, in WRMD, the constellation of paracervical, parascapular, and glenohumeral pain may result from repeated scapular protraction with clerical work that triggers loop entry. Cervical radiculopathy results from degenerative disc diseases producing radiating neuropathy [29] but also features cervicogenic headaches and shoulder weakness and upper back pain [30]. These symptoms may lie outside the domain of cervical root compression. However, they can theoretically be explained by the cervical disc disease relatively weakening the peri-scapular stabilizers except for the PM which maintains its lower trunk input, thus triggering loop entry. Notably, neither injections, decompression, nor fusion provide long-term pain relief on average, suggesting the spinal compression is not the sole pain generator [5,31]. The HDL may be a major source of the residual pain not addressed by spinal intervention.

SAPS encompasses shoulder pain and weakness with overhead reach, with findings of subacromial bursitis and cuff tendinopathy, but the true etiology remains contentious [32]. While imaging and intra-operative findings repeatedly demonstrate cuff tendinopathy, bursitis, and bicipital tendonitis which remain “hard to be explained if not for contact between the rotator cuff and the acromion” [32], current surgeries that alter acromion morphology and reconstruct these structures demonstrate no pain relief on average [6,33,34]. However, the HDL can rectify this paradox, because here the lowering of the acromion and subsequent impingement is driven by the pull of the PM from below. This explains both the repeated findings and the inefficacy of current interventions, as the degraded subacromial structures are secondary [17], while the PM is the true source. TOS’s conventional model of brachial neurovascular compression [4,35,36,37,38,39] does not account for presence of occipital headaches and scapular protraction [40], inefficacy of surgical decompression and scalene chemodenervation [4,30], and higher prevalence in women [30]. But the HDL can explain these as well: the PM protracts the scapula, headaches are a separate loop sequela produced by occipital neuralgia from dorsal trapezius stretch, and the overlying breast in women tightens the PM.

**Figure 7:**
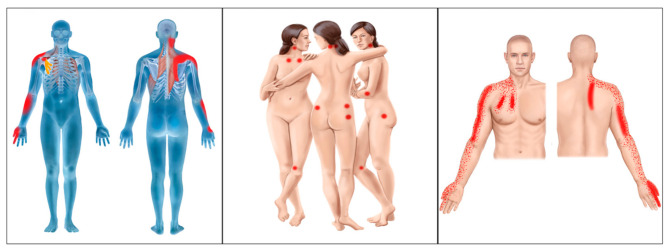
Comparison of HDL to tenderness points in fibromyalgia and trigger points in myofascial pain syndrome. Both MPS (**right**) and fibromyalgia (**center**) feature trigger and tenderness points located at the base of the neck, around the scapula, anterior shoulder, and lateral forearm. This matches the HDL (**left**), which anatomically explains the distribution.

SD has >30 proposed bony, articular, and neurologic causes. Notably, the scapula uniformly protracts [8], but why each cause produces the specific morphology of protraction remains undescribed. Within the HDL, protraction results from a single cause, the direction of pull by the PM, and many of the alleged causes are either sequelae themselves or associations. One form of dyskinesis, SICK Scapula, is theorized to result from the geometry of the scapula gliding “up and over” the ellipsoid thorax, with the PM tightening secondarily [41]. The HDL reverses the causality: PM tightness drives the scapula into dyskinesis via an imbalance of muscular forces rather than bony geometry. PMPS is attributed to neuralgia of the intercostobrachial nerve, but also features neck, upper back, and shoulder pain and radiating neuropathy [7]. These are all HDL sequelae resulting from an initial incitement of plexus neuritis. Both CRPS and fibromyalgia include widespread pain, hand sensorimotor abnormalities, and peri-scapular and peri-occipital trigger points, with no clear cause [42]. However, the HDL produces these symptoms and tenderness/trigger points in the same distribution, but supplies anatomic reasoning (Figure 7).

Moreover, the HDL hypothesizes an evolutionary rationale for the ubiquity of chronic upper limb pain [1,2,3]. Humans are the only obligate bipedal mammal, but our upper limb descends from the quadrupedal forelimb. For quadrupeds, optimal locomotion requires coordination between the forepaw and the ventral scapula stabilizer, so the lower trunk synchronizes both. Protraction is the default scapula position in quadrupeds [43], but pathological in humans [8,15]. Due to an evolutionary idiosyncrasy, our scapula may be prone to reverting to its quadrupedal state. Chronic pain may be the price of our unique shoulder motion, the most of any joint in the mammalian kingdom [13,44]. As acute pain is a signal of tissue damage carrying survival value [12], chronic pain may also have an anatomic basis. This warrants further investigation.

Our study suffers numerous limitations. Chiefly, this is a retrospective study limited to one practice without a control group. Patient selection bias and other confounders, especially the placebo effect, influenced the results [45]. Patients also underwent a regimented pre-operative and post-operative therapy protocol. Hence, the clinical gains cannot be conclusively attributed to the surgery itself. The intent of this study is to hypothesize the HDL’s role in chronic upper limb pain, based on our preliminary results and the theoretical explanations for contemporary gaps in understanding. Nonetheless, our findings must be validated (or refuted) by others. Ideally, the mechanistic aspects of the HDL model should be corroborated by electrodiagnostics and/or advanced imaging. Diagnosis of HDL itself derives from history and physical symptoms, and this accuracy and reproducibility are not yet validated. Neuropathy was diagnosed via the SCT which has its limitations [46], although no gold standard exists [47]. Our principal outcome of pain is subjective, but no objective measurement exists, and self-reported reductions remain meaningful [48]. We did not employ standardized patient-reported outcomes (PROs), and validated instruments such as DASH or SF-36 would better capture impact on overall quality of life [49,50]. Our ~2-year average follow-up does not prove permanence. Crucially, our findings only apply to those ‘in the loop’. The terminal symptoms of the HDL can occur independently. A total of 17% of patients required secondary neurolysis, emphasizing the prevalence of double-crush neuropathy. Surgeons should follow explicit diagnostic criteria (Figure 2), exhaust conservative options first, and closely survey patients after treatment. Breaking the loop via PM+ICN is the first step in a longitudinal and multi-disciplinary process to pain relief.

Pain remains incredibly complex, and collaboration with pain management and other specialties remains essential. The HDL should be seen as a proposed model which may contribute to unexplained chronic pain spanning various disorders, but ultimately needs substantiation via larger, multi-institutional studies that include a control group, utilize PROs, and incorporate objective testing to demonstrate mechanism of action.

## 5. Conclusions

In conclusion, the Human Disharmony Loop—the tendency of the scapula to protract due to its ventral chain’s lower trunk innervation—may constitute a shared anatomic mechanism that contributes to chronic pain across heterogenous disorders of the upper limb girdle. Certain intractable patients may benefit substantially from PM+ICN, but should be counseled that 17% require secondary neurolysis.

## Figures and Tables

**Table 1:** Patient characteristics and clinical presentation.

	*N* = 318 Patients
Age ^1^	51.0 (49.2, 52.9)
BMI ^1^	29.8 (29.0, 30.5)
Gender	Male 101 (31.8%)
	Female 217 (68.2%)
Chronic Pain Syndromes ^3^	Thoracic Outlet Syndrome 53 (16.7%)
	Fibromyalgia 73 (23.0%)
	Complex regional pain syndrome 45 (14.2%)
	Work-related musculoskeletal disorder 60 (18.9%)
	Myofascial pain syndrome 106 (33.3%)
	Cervical radiculopathy 104 (32.7%)
	Burner/Stinger 9 (2.8%)
	Subacromial pain syndrome 266 (83.6%)
	Scapular dyskinesis 13 (4.1%)
	Post-mastectomy pain syndrome 11 (3.5%)
	Cervicogenic headaches 244 (76.7%)
Laterality	Right 186 (58.5%)
	Left 132 (41.5%)
Prior Surgery	Subacromial decompression + adjunct ^2^ 89 (28.0%)
	Total shoulder arthroplasty 27 (8.5%)
	1st rib resection 5 (1.6%)
	Cervical fusion 53 (16.7%)
	Distal neurolysis ^4^ 90 (28.3%)

^1^ Average (95% CI); ^2^ Adjunct procedures include rotator cuff repair, biceps tenodesis, distal clavicle excision, labrum repair; ^3^ Percentages sum to >100 as patients could exhibit multiple diagnoses; ^4^ Includes carpal, cubital, radial tunnel release.

**Table 2:** Clinical outcomes.

	Pre-Operative	Post-Operative	*p*-Value
Pain ^1^	7.3 (7.1, 7.5)	2.1 (1.3, 3)	<0.001
Clinical	Thoracic Outlet 174 (54.7%)	Thoracic Outlet 7 (2.2%)	<0.001
Neuropathy ^2^	Suprascapular 183 (57.5%)	Suprascapular 5 (1.6%)	<0.001
	Axillary 259 (81.4%)	Axillary 51 (16.0%)	<0.001
	Radial 223 (70.1%)	Radial 65 (20.4%)	<0.001
	Cubital 68 (21.4%)	Cubital 64 (20.1%)	0.650
	Carpal 133 (41.8%)	Carpal 51 (16.0%)	<0.001
Headaches	244 (76.7%)	5 (1.6%)	<0.001
ROM ^3^	97 degrees (93, 100)	171 degrees (169, 174)	<0.001
Scapular Dyskinesis ^4^	None 0 (0.0%)	None 294 (92.5%)	<0.001
	Dynamic 4 (1.3%)	Dynamic 18 (5.7%)	
	Static 314 (98.8%)	Static 4 (1.3%)	
Secondary Neurolysis ^5^	N/A	Overall 55 (17.3%)	N/A
		Thoracic Outlet 4 (1.3%)	
		Suprascapular 2 (0.6%)	
		Axillary 30 (9.4%)	
		Radial 31 (9.7%)	
		Cubital 33 (10.4%)	
		Carpal 18 (5.7%)	
Surgical Complications	N/A	Overall 11 (3.5%)	N/A
		Seroma 4 (1.3%)	
		Wound dehiscence 2 (0.6%)	
		Superficial infection 2 (0.6%)	
		Emergent hematoma 1 (0.3%)	
		Revision surgery 1 (0.3%)	
		Rash 1 (0.3%)	

^1^ Average (95% CI); ^2^ Clinical neuropathy on exam was diagnosed via a positive scratch-collapse test at each location.; ^3^ Average active pain-free range of motion (ROM) value for shoulder abduction (95% CI); ^4^ Scapular dyskinesis on exam was classified as: none (no protraction), dynamic (protraction with overhead reach), static (protraction at rest); ^5^ Patients were offered secondary neurolysis for provocative neuropathic lesions on exam causing lingering pain at 3 months postoperatively. N/A = not applicable.

**Table 3:** HDL proposed anatomic explanations for clinical observations of chronic pain syndromes of the upper limb ^1^.

Clinical Observation	Syndrome(s)	HDL Anatomic Explanation
Higher prevalence in women	CRPS, fibromyalgia, TOS	Weight of breast tissue tightens the PM
Persistent and widespread pain not isolated to single nerve distribution disproportionate to any inciting event	CRPS, fibromyalgia	Deformation of the scapula’s connections pathologizes the full girdle from the neck to fingers
Abnormal distal hand sensory, motor, vasomotor, and trophic changes	CRPS, fibromyalgia, TOS	Brachial plexus stretch and costoclavicular compression
Overall poor response to treatment	CRPS, fibromyalgia, TOS, SAPS, MPS, cervical radiculopathy	Positive feedback nature of central loop, and current treatments targeting pathoanatomic sequelae
Trigger or tenderness points of neck, upper back (peri-scapular), anterior shoulder	CRPS, fibromyalgia, MPS, SAPS	Pathological stretch of dorsal scapular muscles, and medial coracoid tenderness
Presence of occipital headaches	Fibromyalgia, TOS, MPS, WRMD, cervicogenic headaches, cervical radiculopathy, PMPS	Occipital neuritis from upper trapezius stretch
Development of trigger points after computer use, piano playing, carrying a backpack, and association of trigger points with cervical spine pathology	MPS, cervical radiculopathy, WRMD	Repeated scapula protraction and/or cervical spine pathology triggers loop entry
Association of neck pain, occipital headaches, and radiating neuropathy	Cervical radiculopathy, cervicogenic headaches	Stenosis of cervical roots triggers loop entry producing upper trapezius stretch, costoclavicular compression and brachial plexus stretch, and occipital neuritis
Scapula protraction	SD, TOS, SICK scapula	PM pulls the scapula in its direction of protraction due its unique C8-T1 innervation

Theoretically, the HDL offers anatomic explanations for some observations seen in chronic pain disorders of the upper limb. This suggests the PM’s effect on the scapula may constitute part of their pathophysiology. ^1^ HDL = Human disharmony loop, CRPS = complex regional pain syndrome, TOS = thoracic outlet syndrome, SAPS = subacromial pain, PMPS = post-mastectomy pain syndrome, SD = scapular dyskinesis, MPS = myofascial pain syndrome, WRMD = work-related musculoskeletal disorder.

## Data Availability

All data has been de-identified and can be accessed at https://doi.org/10.6084/m9.figshare.30994186 (accessed on 1 February 2026).
